# Deep convolutional neural networks for image-based *Convolvulus sepium* detection in sugar beet fields

**DOI:** 10.1186/s13007-020-00570-z

**Published:** 2020-03-05

**Authors:** Junfeng Gao, Andrew P. French, Michael P. Pound, Yong He, Tony P. Pridmore, Jan G. Pieters

**Affiliations:** 1 Lincoln Institute for Agri-food Technology, University of Lincoln, Lincoln, Riseholme Park, LN2 2LG UK; 2 Department of Biosystems Engineering, Ghent University, Coupure Links 653, 9000 Ghent, Belgium; 3 School of Computer Science, University of Nottingham, Jubilee Campus, Wollaton Road, Nottingham, NG8 1BB UK; 4 School of Biosciences, University of Nottingham, Sutton Bonington Campus, Nr Loughborough, LE12 5RD UK; 5 College of Biosystems Engineering and Food Science, Zhejiang University, Yuhangtang Road 866, Hangzhou, 310058 Zhejiang China

**Keywords:** Precision farming, Deep learning, Weed detection, Synthetic images, Transfer learning

## Abstract

**Background:**

*Convolvulus sepium* (hedge bindweed) detection in sugar beet fields remains a challenging problem due to variation in appearance of plants, illumination changes, foliage occlusions, and different growth stages under field conditions. Current approaches for weed and crop recognition, segmentation and detection rely predominantly on conventional machine-learning techniques that require a large set of hand-crafted features for modelling. These might fail to generalize over different fields and environments.

**Results:**

Here, we present an approach that develops a deep convolutional neural network (CNN) based on the tiny YOLOv3 architecture for *C. sepium* and sugar beet detection. We generated 2271 synthetic images, before combining these images with 452 field images to train the developed model. YOLO anchor box sizes were calculated from the training dataset using a k-means clustering approach. The resulting model was tested on 100 field images, showing that the combination of synthetic and original field images to train the developed model could improve the mean average precision (mAP) metric from 0.751 to 0.829 compared to using collected field images alone. We also compared the performance of the developed model with the YOLOv3 and Tiny YOLO models. The developed model achieved a better trade-off between accuracy and speed. Specifically, the average precisions (APs@IoU0.5) of *C. sepium* and sugar beet were 0.761 and 0.897 respectively with 6.48 ms inference time per image (800 × 1200) on a NVIDIA Titan X GPU environment.

**Conclusion:**

The developed model has the potential to be deployed on an embedded mobile platform like the Jetson TX for online weed detection and management due to its high-speed inference. It is recommendable to use synthetic images and empirical field images together in training stage to improve the performance of models.

## Background

Sugar beet (*Beta vulgaris* ssp. vulgaris var. altissima) is very vulnerable to weed competition due to its slow growth and low competitive ability at the beginning of vegetation [1]. The yield loss caused by weed competition can be significant. Therefore, effective weed management in early stages is critical, and essential if a high yield is to be achieved. In modern agriculture, herbicide is widely used to control weeds in crop fields [2]. Weeds are typically controlled by spraying chemicals uniformly across the whole field. However, the overuse of chemicals in this approach has increased the cost of crop protection and promoted the evolution of herbicide-resistant weed populations in crop fields [3], which is a hindrance to sustainable agriculture development.

Site-specific weed management (SSWM) refers to a spatially variable weed management strategy to minimize the use of herbicides [4]. However, the main technical challenge of SSWM implementation lies in developing a reliable and accurate weed detection system under field conditions [5]. As a result, various automated weed monitoring approaches are being developed based on unmanned aerial vehicle or on-ground platforms [6–8]. Among them, image-based methods integrating machine learning algorithms are considered a promising approach for crop/weed classification, detection and segmentation. Previous studies [7] utilized features like shape, texture and colour features with a random forest classifier for weed classification. Others, such as Ahmad el al [9] developed a real-time selective herbicide sprayer system to discriminate two weed species based on visual features and an AdaBoost classifier. Spectral features from multispectral or hyperspectral images could also be exploited for weed recognition [10, 11]. Although the works mentioned above show good results on weed/crop segmentation, classification and detection, challenges such as plant species variations, growth differences, foliage occlusions and interference from changing outdoor conditions still need to be further overcome in order to develop a real-time and robust model in agricultural fields.

Deep learning, a subset of machine learning, enables learning of hierarchical representations and the discovery of potentially complex patterns from large data sets [12]. It has shown impressive advancements on various problems in natural language processing and computer vision, and the performance of deep convolutional neural networks (CNNs) on image classification, segmentation and detection are of particular note. Deep learning in the agriculture domain is also a promising technique with growing popularity. Kamilaris et al. [13] concluded that more than 40 studies have applied deep learning to various agricultural problems like plant disease and pest recognition [14, 15], crop planning [16] and plant stress phenotyping [17]. Pound et al. [18] demonstrated that using deep learning can achieve state-of-the-art results (> 97% accuracy) for plant root and shoot identification and localization. Polder et al. [19] adapted an fully convolutional neural network (FCN) for potato virus Y detection based on field hyperspectral images. Specifically, for crop/weed detection and segmentation, Sa et al. [20, 21] developed WeedNet and WeedMap architectures to analyse aerial images from an unmanned aerial vehicle (UAV) platform. Lottes et al. [8, 22] also did relevant studies on weed/crop segmentation in field images (RGB + NIR) obtained from the BoniRob, an autonomous field robot platform. All these studies have demonstrated the effectiveness of deep learning, with very good results provided.

In practice, farmers usually plow fields before sowing to provide the best chance of germination and growth for crop seeds. Moreover, parts of pre-emergent weeds are buried under the ground and so killed through this procedure. However, *Convolvulus sepium* (hedge bindweed) can emerge from seeds and remaining rhizome segments left underground. This leads to different emergence times of *C. sepium*, resulting in multiple growth stages from first leaves unfolded to stem elongation being represented in a single field. The appearance of *C. sepium* at different growth stages varies. In the early growth stages, some *C. sepium* plants might have similar color features as sugar beet plants in their early growth stages. All these factors bring challenges to the development of a robust system for *C. sepium* detection under field conditions. To the best of our knowledge, no studies have attempted to detect them in a sugar beet field based on a deep learning approach.

In our study, first we develop an image generation pipeline to generate synthetic images for model training. We then design a deep neural network to detect *C. sepium* and sugar beet based on field images. The major objectives of the present study are (i) to appraise the feasibility of using a deep neural network for *C. sepium* detection in sugar beet fields; (ii) to explore whether the use of synthetic images can improve the performance of the developed model; (iii) to discuss the possibility of our model to be implemented on mobile platforms for SSWM.

## Methods

A digital single-lens reflex (DSLR) camera (Nikon D7200) was used to manually collect field images from two sugar beet fields of West Flanders province in Belgium under different lighting conditions (from morning to afternoon in sunny and cloudy weather). Most sugar beet plants have 6 unfolded leaves, while the growth stages of *C. sepium* plants vary widely, from seedling to pre-flowering. The camera was held manually to capture images randomly in the sugar beet fields. The distance between camera and soil surface was around 1 m which is not strictly fixed in order to create more variations in the images. For camera settings, the ISO value is 1600 and the exposure times are 1 ms under sunny weather conditions and 1.25 ms under cloudy weather conditions. The resolution of raw images is 4000 × 6000 pixels. There are 652 images under different lighting conditions which were manually labelled with bounding boxes. Among them, 100 images are randomly selected as a test dataset and 100 images are randomly selected as a validation dataset. The remaining 452 images are used as a training dataset. All the images were resized to 800 × 1200 pixels. In this way, the resized images do not change their aspect ratio and are suitable for training based on our computation resources.

### Synthetic image generation

**Fig. 1:**
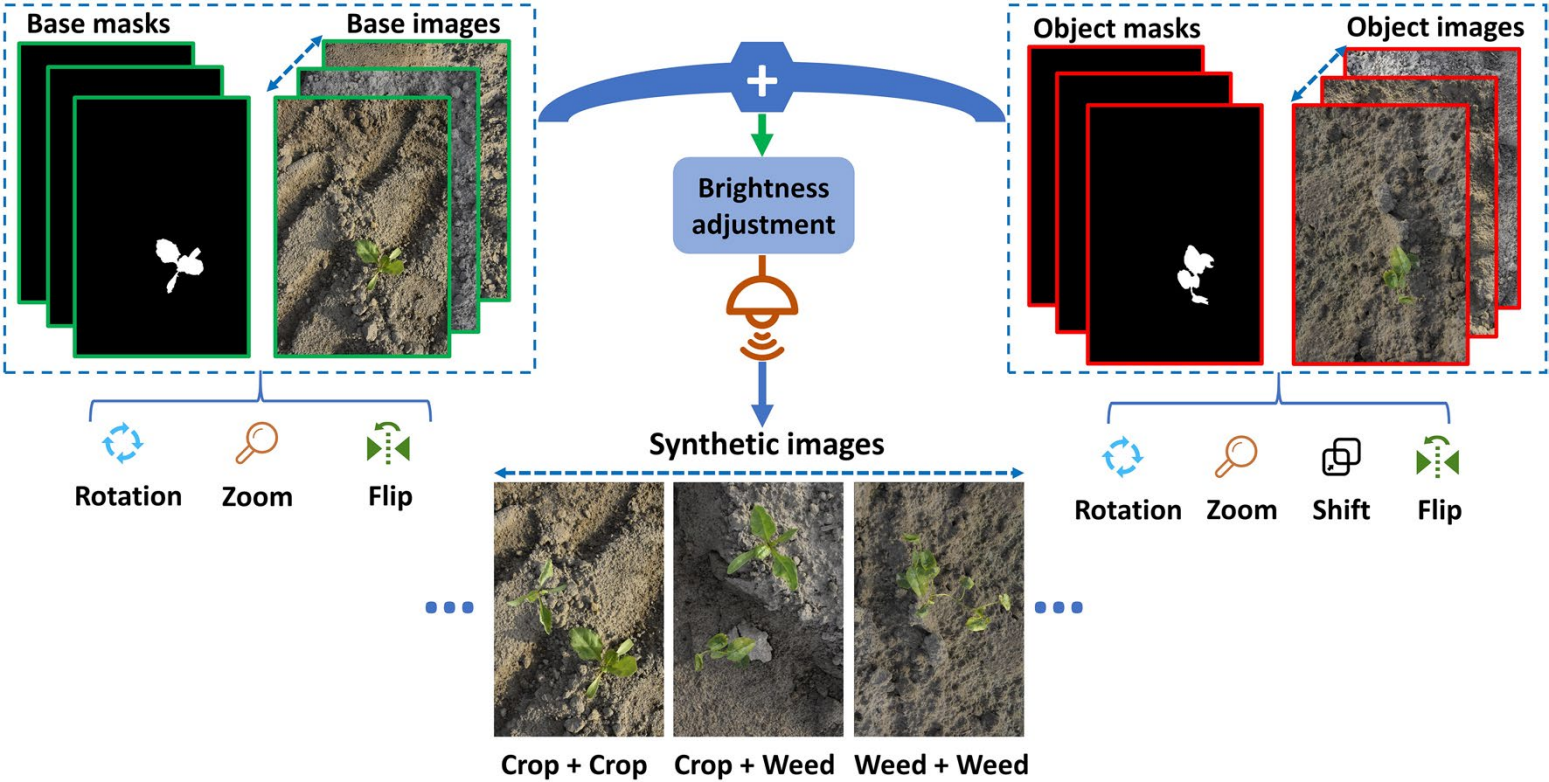
The process of synthetic image generation

**Fig. 2:**
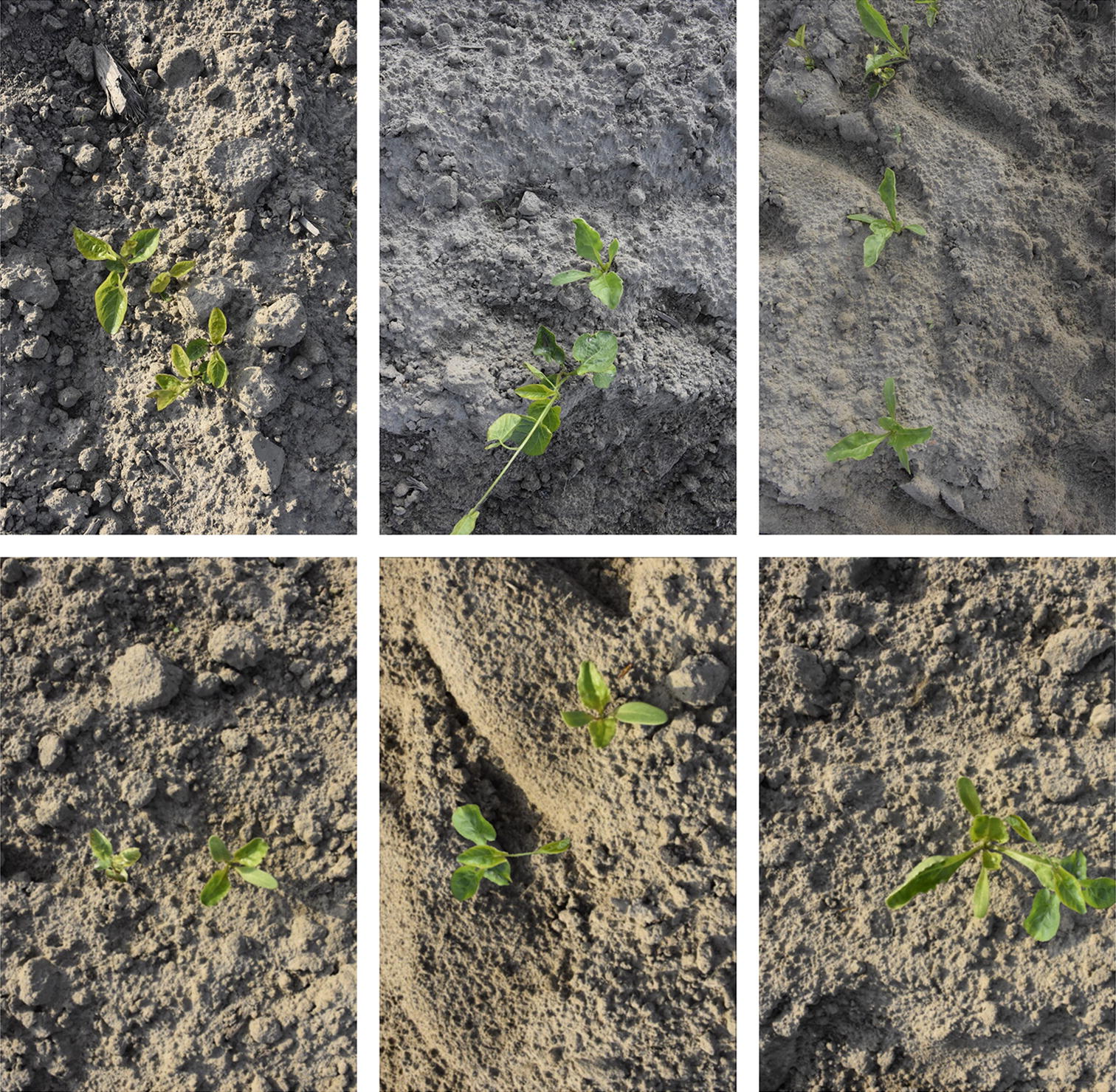
The examples of real and synthetic images (top row: real images, bottom row: synthetic images)

Training a deep neural network with adequate performance generally requires a large amount of data. This is labour-intensive and time-consuming to collect and label. To overcome this problem, we generated synthetic images based on the training dataset from the formerly collected field images. The process of synthetic training image generation is depicted in Fig. 1. Seventy-seven images were selected as original source images. All these images contained either a sugar beet (51) or a *C. sepium* object (26). Their excess green (ExG) vegetation index [23] grayscale images were obtained using Eqs. (1) and (2). Equation (2) is used to normalize R, G and B channel. Next, we converted the ExG grayscale images into binary mask images with Otsu’s algorithm [24]. Afterwards, the object images and their masks were transformed using a set of randomly chosen parameters. Rotation (from 0 to 360° with a 15° step), zoom (from 0.5 × to 1.5 × with 0.1 step), shift (from − 100 to 100 pixels with a 15-pixel step both in the horizontal and vertical directions) and flip (horizontal or vertical direction) operations were applied. The base image and their corresponding masks were subjected to flip (horizontal or vertical direction), limited rotation (0 or 180°) and limited zoom (from 1 × to 1.8 × with 0.1 step) operations to keep the soil background information. The object mask image (Boolean data type) was used as a logic control image. If the logic value in the object mask image is true, the pixel in the base image was replaced by the pixel from the object image. Otherwise, there is no replacement in the base image. After all the pixels from the object images were added to the base images, their brightness was adjusted using Gamma correction [25]. Gamma values varied from 0.5 to 1.5 with 0.2 step. In our study, we generated 2271 synthetic images in total. They are comprised of 1326 (51 × 26) images with sugar beet and *C. sepium* plants, 676 (26 × 26) images with *C. sepium* and *C. sepium* plants and 269 images with sugar beet and sugar beet plants. These synthetic images will be only used for training deep neural networks. The less images (269) with sugar beet and sugar beet plants were generated compared to the other two type images (1326 and 676), because the balance of different object numbers (sugar beet and *C. sepium*) is better to keep for the benefits of training deep neural network after considering most field images only contain sugar beet plants in the training dataset. The examples of real field images and synthetic images are shown in Fig. 2. There is no occlusion in base images and object images. However, the synthetic images could contain overlapped plants (see Fig. 2 bottom right image) as the object (sugar beet or *C. sepium*) was randomly placed in the base images in this pipeline, thus better representing the real scenario of field conditions.

1 $${\text{ExG}} = 2*{\text{g}} - {\text{r}} - {\text{b}}$$

2 $${\text{r}} = \frac{R}{G + R + B},\;{\text{g}} = \frac{G}{G + R + B},\;{\text{b}} = \frac{B}{G + R + B}$$

where R, G and B are the red, green and blue channel pixel values, respectively.

### Deep neural network architecture

**Fig. 3:**
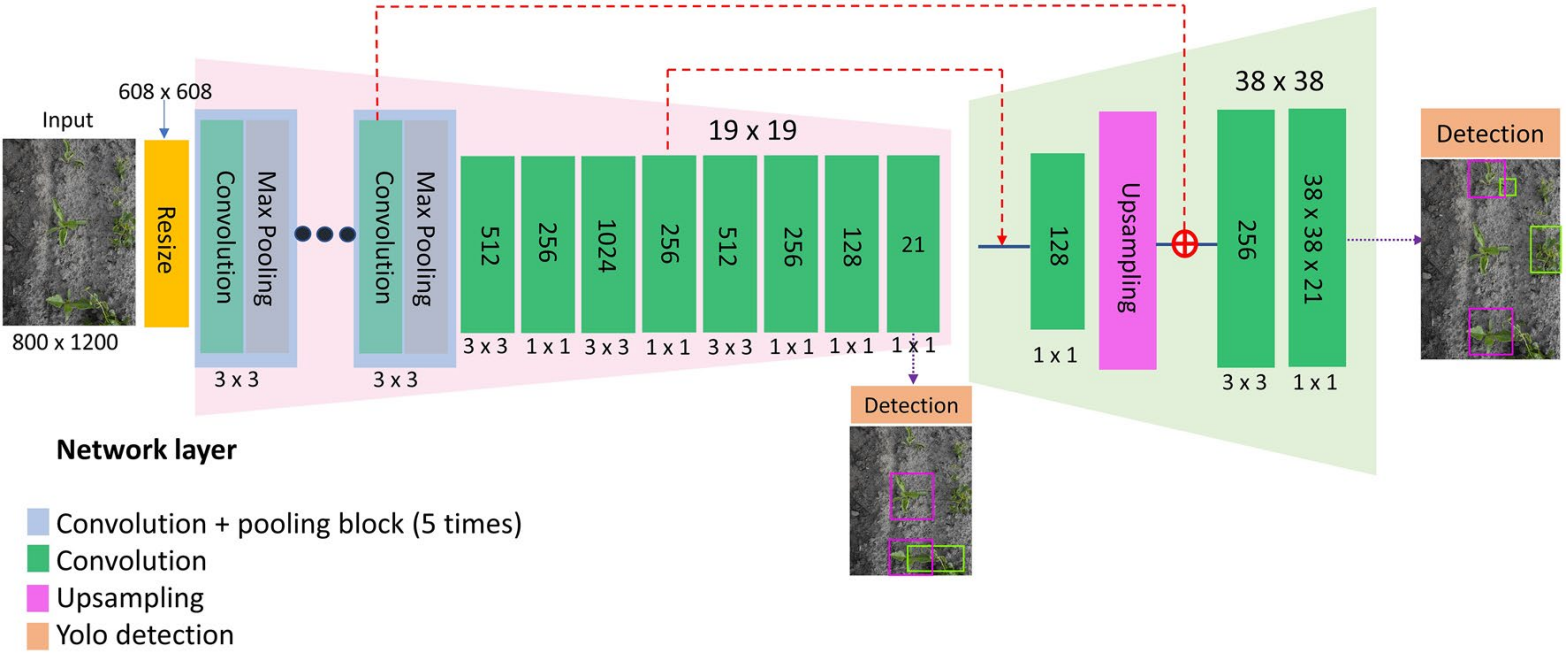
Deep neural network architecture

The deep neural network architecture used in this study is depicted in Fig. 3. It is similar to the tiny YOLOv3 (You Only Look Once) framework, a lighter and faster version of YOLOv3 [26]. In our case, there were only two object classes. The sugar beet objects generally had similar sizes in fields as they were sown in the same time. Thus, we reduced the number of detection scales to two scales instead of three scales in YOLOv3. This change speeded up inference time. Furthermore, we modified the route for feature concatenation and added two more convolutional layers for better feature fusion. Before feeding the image data into networks, all the images were resized to 608 × 608 spatial resolution to fit the network architecture. The network first comprised 5 convolution and max-pooling blocks. The number of convolutional filters in each block, starting with 16 filters, was doubled compared to the former block. The 5 max pooling layers resulted in a total down-sampling by a factor of 32. At the end of convolution and max pooling block, the dimension of the feature map was 19 × 19 × 256. A series of convolution operations were then carried out to obtain the final features (19 × 19 × 21), a 3-dimensional tensor encoding coordinate of the bounding box, object and class predictions, for initial object detection. One of the most notable features of YOLOv3 is to detect objects at different scales. In our network architecture, it detects objects at two different scales with 19 × 19 and 38 × 38 grids, respectively. In the tail of the network, we took the feature map from the previous 15th layer as input for a convolutional layer with 128 filters and then upsampled it by 2×. Subsequently, the upsampled features were concatenated with the earlier feature map resulting from a convolutional layer in the last convolution and max pooling block. Then two more convolutional layers were added to fuse this merged feature map, finally obtaining a similar tensor (38 × 38 × 21) for detection at the second scale. Compared to tiny YOLOv3, we adapted the order of the former layer to concatenate with the upsampled layers to keep the fine grained features and added more layers in decoder part for better fusion low-level features. In our network, instead of using the default anchor box sizes, we calculated our own anchor box sizes based on clustering of object bounding box sizes from the labelled training dataset. K-means clustering [26] approach was used to determine the 6 anchor box sizes for our detection at the two different scales, each scale with 3 anchor boxes. There are three parts, bounding box error for ${L}_{1}$, object confidence error for ${L}_{2}$, and classification error for ${L}_{3}$, in the loss function ${L}_{loss}$ [27]:

$$\begin{aligned}{{L}_{1}} &= {\alpha}_{coord}\sum_{i=0}^{{S}^{2}}\sum_{j=0}^{B}{\omega}_{ij}^{obj}\left[{\left({x}_{i}-{\widehat{x}}_{i}\right)}^{2}+ {({y}_{i}-{\widehat{y}}_{i})}^{2}\right]+ {\alpha}_{coord}\sum_{i=0}^{{S}^{2}}\sum_{j=0}^{B}{\omega}_{ij}^{obj}\left[{\left({\sqrt{w}}_{i}-{\sqrt{\widehat{w}}}_{i}\right)}^{2}+ {({\sqrt{h}}_{i}-{\sqrt{\widehat{h}}}_{i})}^{2}\right] \\ {L}_{2}&=\sum_{i=0}^{{S}^{2}}\sum_{j=0}^{B}{\omega}_{ij}^{obj}{({C}_{i}-{\widehat{C}}_{i})}^{2} + {\alpha }_{noobj}\sum_{i=0}^{{S}^{2}}\sum_{j=0}^{B}{\omega}_{ij}^{noobj}{({C}_{i}-{\widehat{C}}_{i})}^{2}\\ {L}_{3}&=\sum_{i=0}^{{S}^{2}}{\omega }_{i}^{obj}\sum_{c\in classes}{({p}_{i}(c)-{\widehat{p}}_{i}(c))}^{2} \\ {L}_{loss}&= {L}_{1}+{L}_{2}+{L}_{3} \end{aligned}$$

where the weight constants ${\alpha }_{coord}$, ${\alpha }_{noobj}$ are 5 and 0.5, respectively. ${\alpha }_{coord}$ is ten times of ${\alpha }_{noobj}$ in order to focus more on detection. *S* is the number of the grid cell and *B* is the number of bounding box at each scale. ${\omega }_{ij}^{obj}$ denotes that the *j*th bounding box in the grid cell *i* is responsible for this prediction. The value is 1 if there is an object in cell and 0 otherwise. ${\omega }_{ij}^{noobj}$ is the opposite of ${\omega }_{ij}^{obj}$. c is the classes. ${\omega }_{i}^{obj}$ is 1 when the particular class is predicted, otherwise the value is 0.${x}_{i}$*,*${y}_{i}$*, *${w}_{i}$ and ${h}_{i}$ are the centroid coordinate, width and height of the corresponding responsible anchor box. ${C}_{i}$ is the confidence score of object ${p}_{i}(c)$ is the classification loss. The parameters with hats are the corresponding estimated values.

### Transfer learning

Transfer learning uses partial weights from a pre-trained model on a new problem to overcome any potential overfitting due to the lack of sufficient training data. It has been demonstrated that the first layer of deep neural networks extracts some generic features like edge and colour features [28] so that they are generally applicable to other computer vision tasks. Therefore, weights from these layers are expected to be more valuable when optimising the algorithm than randomly initialized weights in the networks [29]. In our study, we used the weights from the pre-trained model (darknet53), trained on the ImageNet dataset, a public dataset containing millions of natural images, to train the proposed, Tiny and YOLOv3 models. The Adam optimizer [30] with the initial learning rate 0.02, then dropping this value by 0.1 at every 20,000 iterations, was chosen to minimize the loss function. The batch size was set to 64. Data augmentation such as random scaling and cropping, and randomly adjusting exposure and saturation was also used during all the training process to reduce the risk of overfitting.

### Evaluation metrics

For object detection applications, mean average precision (mAP) is a standard metric for evaluation of model performance. In our case, we calculated the average precision (AP) of sugar beet and *C. sepium* classes separately, and then averaged over APs of these two classes to calculate mAP value (Eq. 5) to check the overall performance of a model. Precision is a ratio of true object detections to the total number of objects that a model predicted. Recall is a ratio of true object detections to the total number of objects in the dataset. In our case, to be the true object detections, the area of the overlap, also called intersection over union (IoU, Eq. (3)), between the predicted bounding box and ground truth bounding box should exceed 0.5. The AP, calculated by Eq. 4, is precision averaged across all the values of recall between 0 and 1, namely the area under the PR curve [31]. An approximation of the area is calculated via Riemann summation. Note that both precision and recall metrics vary with IoU thresholds. In our case, we set the model threshold as 0.5 (at IoU = 0.5) and then combine all the detections from all the test images to draw a precision-recall (PR) curve. mAP_50_ and AP_50_ denote that the two values were achieved under the condition of IoU = 0.5. 

3 $${\text{IoU}} = \frac{{areaB_{p} \cap areaB_{gt} }}{{areaB_{p} \cup areaB_{gt} }}$$

where $area{B}_{p}$ is the area of the predicted bounding box, and $area{B}_{gt}$ is the area of ground truth bounding box.

4 $${\text{AP}} = \sum\limits_{k = 1}^{N} {P(k)} \Delta recall(k)$$

5 $${\text{mAP}} = \frac{{\sum\limits_{m = 1}^{M} {AP(m)} }}{M}$$

where $N$ is the total number of images in the test dataset,$M$ is the number of classes, $P\left(k\right)$ is the precision value at $k$ images and $\Delta recall(k)$ is the change of the recall between $k$ and $k$ − 1 images.

## Results

### Model performance

**Fig. 4:**
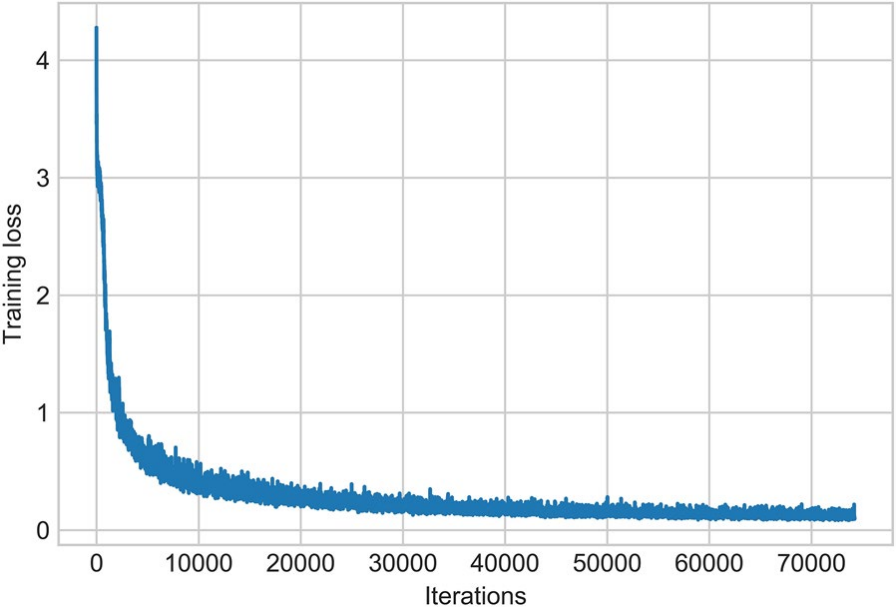
Loss curve of the proposed detection network

**Fig. 5:**
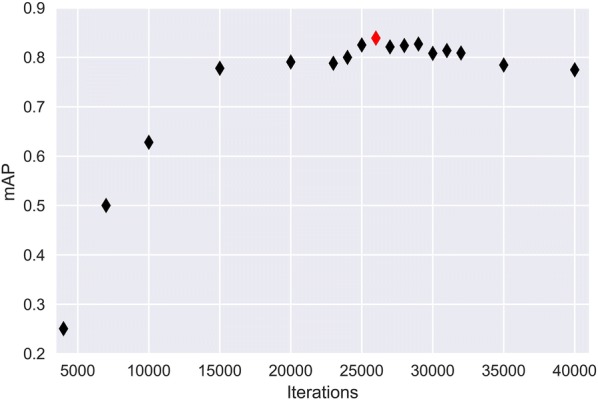
mAP_50_ values of the developed model in the validation dataset at different batch iterations

The training loss curve of the proposed deep neural network is shown in Fig. 4. As we can see, the training loss dropped sharply at the beginning of the training stage, and then the loss value slowly converged at around 0.18 after 22,000 batch iterations (527 epochs). We evaluated the performances of the developed model in the validation dataset at different batch iterations (Fig. 5). It is shown that the mAP_50_ obtained the highest value (0.839) in 26,000 iterations. After 26,000 iterations, the mAP_50_ started to slowly decrease as the model tends to be overfitting in the validation dataset, though the training loss still drops a little. We used the weights (26,000 iterations) to evaluate the developed model in the test dataset. Following the same procedure to other models, Table 1 sums up the performances of the other networks in the test dataset. In general, the proposed network achieved the highest average precision (AP_50_) of *C. sepium* detection (0.761). Although the YOLOv3 obtained the highest mAP_50_ (0.832) and the maximum AP_50_ value of sugar beet (0.938), it did not show good capability in *C. sepium* detection (0.726), which is the priority and most important consideration in SSWM.

**Table 1:** Detection performances of the different models in the test dataset

Model	Average inference time (ms)	mAP_50_	*C. sepium* AP_50_	Sugar beet AP_50_
YOLO V3	40.75	*0.832*	0.726	*0.938*
YOLO V3-tiny	*6.39*	0.810	0.705	0.914
Proposed	6.48	0.829	*0.761*	0.897

Italic values indicate the best values compared to others

**Fig. 6:**
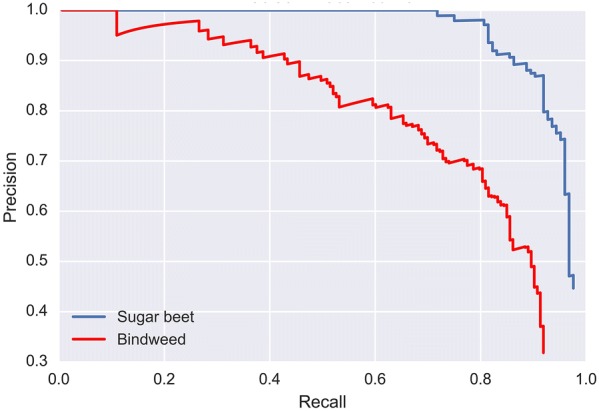
Precision-recall curves of sugar beet and *C. sepium* (bindweed) in the proposed network

**Fig. 7:**
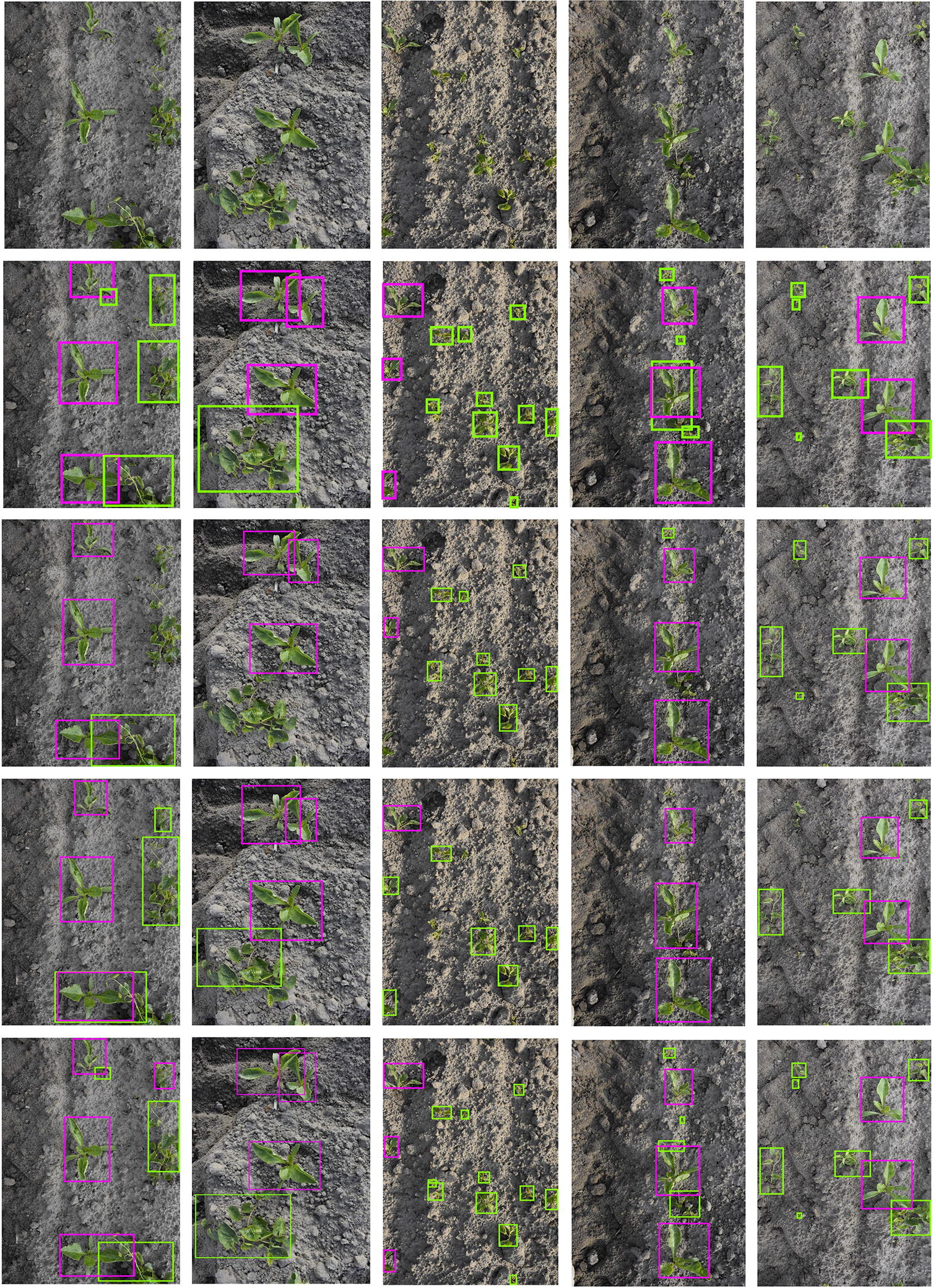
Detection results comparison in the test dataset. From top to bottom, the first row is the input images. The second row is the ground truth of the input images, the third row is detection results from the YOLOv3, the forth row is detection results from the tiny YOLOv3 and the last row is the detection results from the proposed networks

In terms of averaged inference time, all the trained networks were tested on a Linux server with an NVIDIA Titan X Pascal GPU (12G memory). The YOLOv3 model cost on average 40.75 ms to predict an 800 × 1200 image in test data. However, the tiny YOLOv3 and the proposed network performed much faster predictions, with detections in the same spatial resolution images at 6.39 ms and 6.48 ms, respectively. This can be attributed to the use of a less deep network architecture, thus the number of parameters needed to be tuned were far fewer than the YOLOv3 network. Figure 6 displays the precision-recall curves of sugar beet and *C. sepium* of the proposed network in the test dataset. The specific detection results of these three networks in 5 typical field images are provided in Fig. 7.

### Synthetic images

**Fig. 8:**
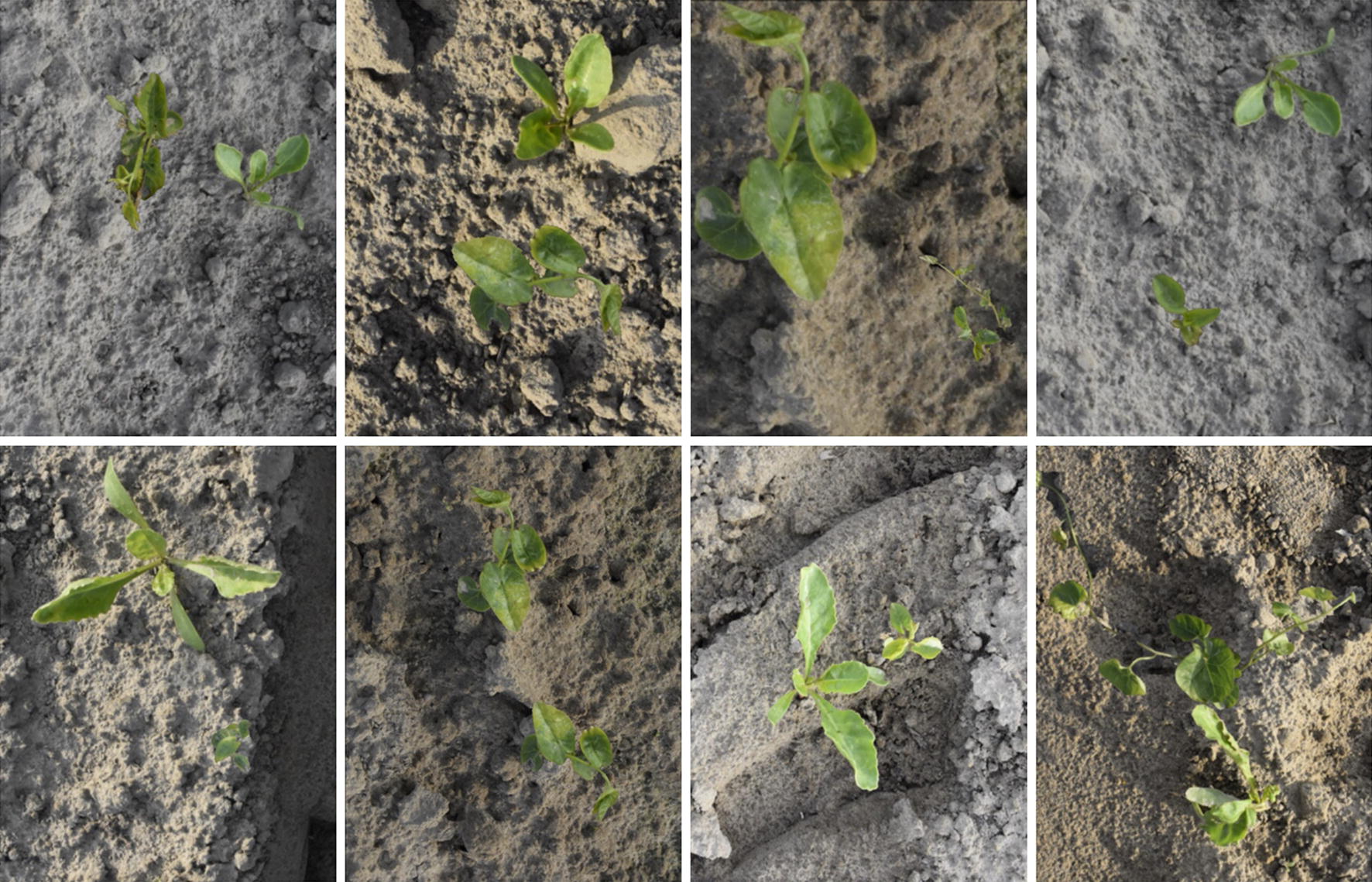
Examples of the synthetic images

In this study, we used 2271 images for training the models and some examples are given in Fig. 8. Table 2 displays the effect of adding synthetic images. It can be seen that the overall mAP_50_ metric increased from 0.751 to 0.829 with the added synthetic images. The contributions come from the improvement of *C. sepium* detection increasing from 0.587 to 0.761.

**Table 2:** Detection results with the different training dataset

Training data	mAP_50_	*C. sepium* AP_50_	Sugar beet AP_50_
Original field images	0.751	0.587	*0.915*
Synthetic images	0.698	0.504	0.891
Original and synthetic images	*0.829*	*0.761*	0.897

Italic values indicate the best values compared to others

### Anchor box

The default anchor box sizes in the tiny YOLOv3 model were [10, 14], [23, 27], [51, 34], [81, 82], [135, 169] and [334, 272]. We used k-means clustering to calculate the 6 anchor box sizes based on our own training data set. The 6 anchor box sizes used for training are [14, 20], [32, 38], [56, 40], [75, 90], [185, 168] and [364, 222], respectively. The effect of anchor box for training is given in Table 3. We can see that the detection results (mAP_50_ = 0.829) with the own calculated anchor box sizes are slightly better than the results (mAP_50_ = 0.823) from the default anchor box sizes.

**Table 3:** Detection results from different Anchor box size sets

Anchor box size	mAP_50_	*C. sepium* AP_50_	Sugar beet AP_50_
Default	0.823	0.756	0.890
Own calculated	0.829	0.761	0.897

### Discussion

Transfer learning with adaptive learning rates was used to train our neural network, leading the training loss to sharply decrease at the beginning, before finally converging at a low loss value. In terms of weight initialization, the experiment [29] has shown that initializing the deep learning models with pre-trained weights from ImageNet leads to better accuracy in many cases. When training a deep neural network, data is a crucial component to reduce the risk of overfitting. We generated more than 2000 synthetic images for training based on conventional image processing techniques. Previous studies [32, 33] have presented other approaches to generate images for object detection and segmentation. Moreover, Generative Adversarial Networks (GANs) [34], inspired by game theory, is also a promising deep learning based approach to generate synthetic images for training neural networks [35]. Open source rendering software such as Blender [36] could be employed to generate synthetic images from 3D models [37]. Back to our approach for synthetic images generation, several ways can be done for improving the quality of synthetic images. For example, the selected base images and object images were taken under same view and lighting conditions. This could assure that the added objects fit well in the background of base images.

Small object detections can be a very challenging problem, especially when using deep neural networks with pooling layers, due to the loss of spatial resolution. Increasing resolution of the input images is a direct way to alleviate that problem but it is usually constrained by the network architecture used and computation resources available. The proposed network detects objects at two different scales. The second scale is capable of detecting small objects because the feature maps are upsampled and then concatenated with the previous feature map, which contains fine-grained features for small object detections. Besides, Ren et al. [38] adapted Faster-RCNN for small object detection in remote sensing images. We also find that the detection results were improved by generating synthetic images based on conventional image processing techniques.

**Fig. 9:**
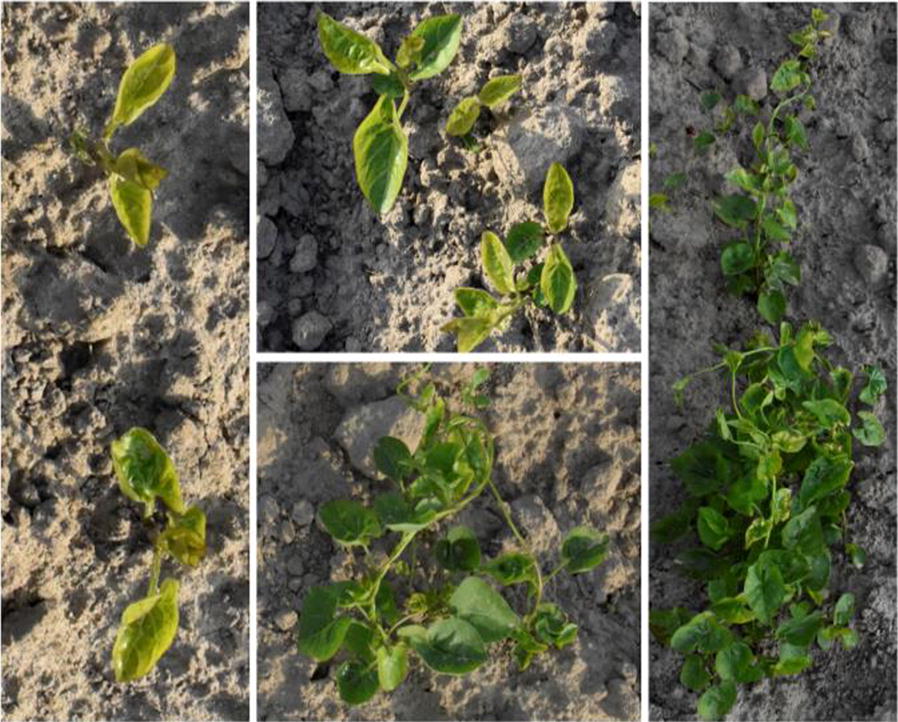
*C. sepium* representations in the field

Under field conditions, most sugar beets generally present relatively homogeneous appearances as their seeds were sown at the same time. However, *C. sepium* can present significant differences in colour, size, morphology and texture. Thus, the variations of *C. sepium* plants shown in Fig. 9 are far more than sugar beet crop. This is likely why all the networks in our study provide better sugar beet detection than *C. sepium*. For row crops like sugar beet, maize and potato, inter-row weeds can be detected after crop line detection [7]. These detected inter-row weeds have the potential to be used as training samples for intra-row weed detections. Kazmi et al. [39] used conventional image processing algorithms and explored hand-crafted features with traditional machine learning techniques for creeping thistle weed detection in sugar beet fields. Although good accuracy was achieved with only using colour information, the use of hand-crafted features makes it difficult to guarantee the robustness of the developed model under changing environmental conditions and variations in plant development. In contrast, deep learning methods can extract hierarchical features and learn very complex functions with a large amount of data provided [12]. Detecting weeds under different weather conditions is not the main challenge when using deep learning-based models because of the use of data augmentation such as random exposure and saturation adjustments or training with a large number of images collected in different weather conditions. It is more difficult to accurate detect weeds under heavy overlapping, and variable object size and shape scenarios. Suh et al. [40] discussed the classification of sugar beet and volunteer potato under field conditions using a VGG-19 modified neural network. A classification accuracy of 98.7% (inference time less than 0.1 s) was obtained, which exceeded previously reported accuracies by Nieuwenhuizen et al. [41] and Suh et al. [42] with hand-crafted features and conventional machine learning algorithms. However, the proposed approach [40] did not lead to the precise detection of volunteer potato in field images because it is a classification task without detecting individual plants in crop fields. The approach used in this study is capable of detecting *C. sepium* plants of various sizes. Compared to other studies [8, 19, 22] that used a hood or artificial lighting for image acquisition, our study targets weed detection under uncontrolled environments. It is difficult to directly compare the performance of the developed model as different datasets and metrics are used in different studies. To the best of our knowledge, this study is the first to detect *C. sepium* in sugar beet fields. Though a proper comparison is lacking, it seems fair to claim that deep learning-based *C. sepium* detection can be made under field conditions.

The own-calculated anchor boxes do not show much improvement in mAP_50_ metric compared to the default bounding boxes which can be attributed to relatively small differences between calculated and default anchor boxes and only 2 object classes in our case. However, it is still highly recommended to use a k-mean clustering approach to calculate prior anchor box sizes for network. It would give networks the range of predicted bounding boxes for most objects, leading to a more accurate bounding box prediction. In practice, the choice of a network depends on a trade-off between accuracy and inference speed. The proposed network achieves a good balance with speed and accuracy. The proposed network shows a sizeable advantage in inference speed at only 6.48 ms per image. One of the reasons for this is the use of a shallower network architecture compared to the Darknet-53 based YOLOv3 [26] and VGG based Single Shot MultiBox Detector (SSD) architectures [43]. Another reason is the mechanism of employing anchor boxes, which does not require a computationally expensive region proposal step when selecting potential object candidates, as regional convolutional neural networks (R-CNN) [44] do. Although the test is performed on a desktop computer with an NVIDIA Titan X GPU, it is still possible to be implemented on real-time systems with a state-of-the-art mobile embedded device like NVIDIA Jetson TX [45].

The DSLR Nikon camera provides high spatial resolution raw images (4000 × 6000) for field data collection. In this study, the original images were resized twice to 608 × 608 pixels before feeding into networks. In this aspect, it is not necessary to use a very high resolution and costly imaging sensor when developing a vision-based site-specific spraying field robot with the trained deep neural network model. An affordable webcam is probably suitable for this prototype development as it also meets the resolution requirement and it is easy to use and low-cost. In this work, the synthetic images contain two objects (weeds or crop), which is still not complex enough compared to true field images, despite some overlapped plants images generated. More challenging synthetic images thus need to be introduced for training the networks in order to represent near-true harsh field conditions. Besides, we only investigated the effect of 2271 synthetic images for training networks without consideration of other number of synthetic images due to limited number of base and object images. It would be helpful to compare results among other number of synthetic images (e.g. 3000, 4000, 5000) to determine the optimal number of synthetic images for training neural networks. Barth et al. [46] discussed the effects of synthetic data size for model performances. Furthermore, it is interesting to investigate which crop growth stages result in the optimal prediction results.

Our object detection results are denoted as bounding box formations. The coordinate information of bounding boxes in the image could be used to estimate actuator action time in the real world when developing a target spray platform with a machine vision system. Other than field vehicle platforms for weed management, drone-based platforms are also gaining popularity for weed mapping in precision farming [7, 21]. To put this study into perspective, the future works will be done on SSWM prototype development based on the deep learning algorithms. Besides, pixel-wise crop/weed segmentation based on fully convolutional networks (FCNs) is also worthwhile to be explored as it provides more precise predictions on decision boundary compared to object detections with bounding boxes. In terms of synthetic data, other ways like using GANs will be explored as well in the future.

## Conclusion

In this paper, we developed a pipeline to generate synthetic images from collected field images. There were 2271 synthetic images and 452 field images in total for training. Moreover, we designed a deep neural network based on the tiny YOLO architecture for *C. sepium* and sugar beet detection. We recommend calculating anchor box sizes based on an application-specific dataset instead of using the default values when employing YOLO-based neural networks. The added synthetic images in the training process improved the performance of the developed network in *C. sepium* detection. Comparing to other networks like YOLOv3, we conclude that our network achieved a better trade-off between speed and accuracy. Specifically, the average precisions (AP_50_) of *C. sepium* and sugar beet were 0.761, 0.897, respectively with 6.48 ms inference time per image (800 × 1200) on an NVIDIA Titian X GPU environment. The trained model could be deployed in a mobile platform (e.g., unmanned aerial vehicles and autonomous field robots) for weed detection and management. Finally, based on the speed and accuracy results from our network, we believe that the advancement of new deep learning architecture and mobile computing device, together with a large amount of field data will significantly contribute the development of precision agriculture like site-specific weed management (SSWM) in the coming years.

## Data Availability

Data is available on request to the authors. The trained deep neural network weight is uploaded at https://drive.google.com/file/d/1-E_b_5oqQgAK2IkzpTf6E3X1OPm0pjqy/view?usp=sharing.

## Abbreviations

CNN: Convolutional neural network
mAP: Mean average precision
SSWM: Site-specific weed management
FCNs: Fully convolutional networks
UAV: Unmanned aerial vehicle
DSLR: Digital single-lens reflex
ExG: Excess green
YOLO: You only look once
IoU: Intersection of union
*C. sepium*: *Convolvulus sepium*
